# The presence of polymorphisms in genes controlling neurotransmitter metabolism and disease prognosis in patients with prostate cancer: a possible link with schizophrenia

**DOI:** 10.18632/oncotarget.27921

**Published:** 2021-03-30

**Authors:** Gennady M. Zharinov, Sergei E. Khalchitsky, Alexandre Loktionov, Marina V. Sogoyan, Yulia V. Khutoryanskaya, Natalia Yu. Neklasova, Oleg A. Bogomolov, Ilya V. Smirnov, Marina P. Samoilovich, Vladimir N. Skakun, Sergei V. Vissarionov, Vladimir N. Anisimov

**Affiliations:** ^1^A.M. Granov Russian Research Center for Radiology and Surgical Technologies of the Ministry of Health of the Russian Federation, Pesochny, St. Petersburg, 197758, Russia; ^2^H. Turner National Medical Research Center for Children's Orthopedics and Trauma Surgery of the Ministry of Health of the Russian Federation, Pushkin, St. Petersburg, 196603, Russia; ^3^DiagNodus Ltd, Babraham Research Campus, Cambridge, CB22 3AT, United Kingdom; ^4^St. Petersburg State Pediatric Medical University of the Ministry of Health of the Russian Federation, St. Petersburg, 194100, Russia; ^5^Yaroslav-the-Wise Novgorod State University of the Ministry of Science and Higher Education of the Russian Federation, Veliky Novgorod, 173003, Russia; ^6^N.N. Petrov National Medical Research Center of Oncology, Pesochny, St. Petersburg, 197758, Russia; ^*^These authors contributed equally to this work

**Keywords:** prostate cancer, prostate-specific antigen, disease prognosis, neurotransmitters metabolism genes, psychiatric disorders

## Abstract

Polymorphisms of neurotransmitter metabolism genes were studied in patients with prostate cancer (PC) characterized by either reduced or extended serum prostate-specific antigen doubling time (PSADT) corresponding to unfavorable and favorable disease prognosis respectively. The ‘unfavorable prognosis’ group (40 cases) was defined by PSADT ≤ 2 months, whereas patients in the ‘favorable prognosis’ group (67 cases) had PSADT ≥ 30 months. The following gene polymorphisms known to be associated with neuropsychiatric disorders were investigated: a) the STin2 VNTR in the serotonin transporter *SLC6A4* gene; b) the 30-bp VNTR in the monoamine oxidase A *MAOA* gene; c) the Val158Met polymorphism in the catechol-ortho-methyltransferase *COMT* gene; d) the promoter region C-521T polymorphism and the 48 VNTR in the third exon of the dopamine receptor *DRD4* gene.

The STin2 12R/10R variant of the *SLC6A4* gene (OR = 2.278; 95% CI = 0.953–5.444) and the -521T/T homozygosity of the DRD4 gene (OR = 1.579; 95% CI = 0.663–3.761) tended to be overrepresented in PC patients with unfavorable disease prognosis. These gene variants are regarded as protective against schizophrenia, and the observed trend may be directly related to a reduced PC risk described for schizophrenia patients. These results warrant further investigation of the potential role of neurotransmitter metabolism gene polymorphisms in PC pathogenesis.

## INTRODUCTION

The importance of complex networks of heterotypic interactions between multiple distinct cell types (both malignant and normal) and regulatory circuits has now become widely recognized. It is also accepted that under certain (still poorly understood) circumstances these diverse components may assemble and collaborate to produce neoplasms and lead to cancer progression [1, 2]. These advances provoked a gradual paradigm shift regarding views on carcinogenesis in general. The traditional tumor-centric view focused exclusively on malignant cell populations has largely been replaced with a concept of tumor microenvironment (TME), the latter being regarded as a “dynamic interaction arena in which tumor cells interact with the extracellular matrix (ECM), resident and recruited cells, and soluble factors” [3]. Indeed, tumor-TME crosstalk involves such major phenomena as metabolism profiling, a range of immune responses (including inflammation), ECM remodeling, and angiogenesis; concerted action of these components modulates tumor growth [2–7]. However, the current fixation of many investigators on the TME may create a bias of exaggerating the importance of immediate surroundings of neoplastic cells in affecting cancer progression, whereas more remote layers of tumor environment within the organism are sometimes overlooked [8].

It is well established that cancer is a disease driven by multiple genetic changes occurring in the malignant cells [1]. At the same time, numerous polymorphic gene variants commonly present in the human genome were shown to mildly influence cancer pathogenesis and tumor progression [9, 10], and it appears that that this genetic variability may contribute to occasionally detected associations between malignancies and non-oncological conditions. One of such associations was highlighted by repeated observations of liaisons between emotional stress, depression and chronic psychiatric disorders with the risk of cancer development [11–16]. Although there is a general consensus that chronic stress and depression tend to result in an impairment of the immune responses and might facilitate cancer initiation and progression [11, 14, 15], the risk of developing some cancers appeared to be decreased in patients with schizophrenia [13, 16]. Remarkably, a significantly reduced risk of developing prostate cancer (PC) was consistently reported for men suffering from this severe condition [13, 16–18].

PC is not only the second most common cancer in men [19], but this is also a condition characterized by a wide variation of severity that ranges from indolent to highly aggressive disease [20]. The latter feature of PC makes tumor growth monitoring a very important prerequisite for successful disease management, and repeated measurement of the concentration of a blood biomarker of PC, prostate-specific antigen (PSA), is generally accepted as an important prognostic tool for routinely monitoring patients with this condition [21–25]. Over the past 25 years, more than 6,000 PC cases were observed and treated at the Russian Scientific Center of Radiology and Surgical Technologies (RSCRST – St Petersburg, Russia). The results of the primary examination and consequent follow-up of these patients allowed creating the “Database of Prostate Cancer Patients” registered in Russia in 2016 [26]. The information on PSA concentration dynamics before treatment was available for about 2,500 of the cases included in the database, making it possible to determine the doubling time of PSA concentration (PSADT) that allows assessing tumor growth rate and aggressivity, serving as a valuable prognostic criterion [23–25]. It is also remarkable that the psychological state of the PC patients included in the database appeared to correlate with the PSADT and disease prognosis [27], thus corroborating observations of other authors mentioned before [11–18].

It is well established that many principal traits of an individual’s temperament, character, and emotions are genetically determined, being controlled by the products of neurotransmitter-encoding genes that are often highly polymorphic. Polymorphisms present in the genes controlling mediators of the serotonin and dopamine systems are known to modulate predispositions to aggressiveness, depression, bipolar disorder, schizophrenia, suicidal and deviant behavior in general [28–32]. The presence of such polymorphisms may entail gene expression changes that often result in modifications of important metabolic patterns that can potentially interfere with neoplasia development, but this intriguing area remains poorly investigated.

It was noted above that psychiatric disorders may be associated with a seriously altered PC risk, but little is known about possible influences of patients’ genetic background both on this phenomenon and on disease prognosis when PC is already present. The aim of this small pilot study was to compare the presence of common polymorphic variants of a few genes encoding neurotransmitters in PC patients with the ‘polar’ variants of disease prognosis defined by either very low or very high PSADT values.

## RESULTS

### Clinical characteristics of study participants

General clinical characteristics of PC patients included in the study are presented in Table 1. The table clearly shows that the men in the unfavorable prognosis group were characterized by significantly higher (*P* < 0.001) Gleason scores, significantly higher (*P* < 0.001) initial PSA concentrations, and significantly lower (*P* < 0.001) PSADT values. It is also evident that men with low PSADT values (favorable prognosis group) had a much longer pre-treatment period (*P* < 0.001), sometimes reaching a few years. At the same time, no age difference between the groups could be detected.

**Table 1:** Clinical characteristics of PC patients included in the study

Index	Disease prognosis (according to PSADT)		*P*-value
	Unfavorable (low PSADT)	Favorable (high PSADT)	
Number of patients	40	67	-
Age at the time of diagnosis, years Median (IQR^*^)	66.4 (61.5–70.7)	68.0 (63.0–72.6)	> 0.05
Gleason score Mean (95% CI^**^)	7.7 (7.5–8.0)	6.4 (6.2–6.5)	< 0.001
Initial PSA, ng/ml Median (IQR)	109.0 (41.6–328.9)	12.9 (9.6–18.7)	< 0.001
Duration of baseline PSA history before treatment, months	2.0 (1.0–3.0)	59.0 (29.0–110.5)	< 0.001
Number of initial analyzes for PSA before treatment	2.0 (2.0–2.0)	10.0 (4.0–20.0)	< 0.001
PSADT, month, Median (IQR)	1.4 (0.8–2.0)	77.1 (49.6–119.0)	< 0.001

^*^IQR – interquartile range. ^**^CI – confidence interval.

### Patient genotyping results

#### *SLC6A4* gene

The presence of polymorphic variants of the *SLC6A4* gene encoding serotonin transporter is known to be associated with psychiatric disorders [28, 29]. Table 2 shows the results of STin2 VNTR (variable number of tandem repeats) genotyping in the second intron of this gene in PC patients with unfavorable and favorable disease prognosis. The STin2 polymorphism is a region of variable repeats in intron 2 and includes two major alleles “STin2.10” and “STin2.12”, which correspond to 10 or 12 repeats (10R or 12R) of 17 bp respectively. In addition, there are less frequent “STin2.7” and “STin2.9” alleles corresponding to seven and nine repeats respectively. Table 2 demonstrates that differences between the compared groups were observed for the genotypes 12R/12R, 12R/10R, 10R/10R, as well as for the homozygosity, and heterozygosity at this locus in general. In particular, the 12R/12R and 10R/10R variants and homozygosity at the locus appeared to be associated with a better PC prognosis, whereas the 12R/10R genotype and heterozygosity at the locus were associated with a poorer PC prognosis. Although these differences between the groups failed to reach statistical significance, the results for the 12R/10R variant, homozygosity and heterozygosity were only marginally non-significant. It should also be noted that in some cases genotyping could not be performed due to poor sample quality, thus the number of analyzed patients was strictly limited.

**Table 2:** Genotype frequencies for the STin 2 VNTR polymorphism of the SLC6A4 gene in groups of PC patients defined as ‘polar opposites’ prognostically

Genotypes for *SLC6A4* STin2 VNTR polymorphism, number of 17 bp repeats	Disease prognosis (according to PSADT)				Odds Ratio (95% CI^*^)	*P*-value
	Unfavorable (low PSADT)		Favorable (high PSADT)			
	*n*	%	*n*	%		
12R /12R	4	11.76	13	22.03	0.472 (0.140–1.584)	0.224
12R/10R	17	50,00	18	30.51	2.278 (0.953–5.444)	0.064
12R/9R	1	2.94	1	1.69	1.758 (0.106–29.036)	0.693
12R/7R	0	0	1	1.69	0.565 (0.022–14.263)	0.729
10R/10R	6	17.65	16	27.12	0.576 (0.201–1.649)	0.304
10R/9R	5	14.71	7	11.86	1.281 (0.373–4.401)	0.694
10R/7R	1	2.94	2	3.39	0.864 (0.075–9.893)	0.906
9R/7R	0	0	1	1.69	0.565 (0.022–14.263)	0.304
*Homozygosity*	*10*	*29.41*	*29*	*49.15*	*0.431 (0.176–1.057)*	*0.066*
*Heterozygosity*	*24*	*70.59*	*30*	*50.85*	*2.320 (0.946–5.690)*	*0.066*
Total	34^**^	100.0	59^**^	100.0	-	-

^*^CI – confidence interval. ^**^Analyzed patient numbers are lower than group totals as genotyping failed in some cases. *Additional genotype groupings are italicized*.

#### *MAOA* gene

The *MAOA* gene encoding monoamine oxidase A (MAOA) and located on the short arm of the X chromosome contains a VNTR of 30bp with 2, 3, 3.5, 4, or 5 repeated copies [33]. The most common variants are alleles with three and four repeats, the latter one being responsible for a higher activity of the enzyme [33]. Conversely, the presence of the allele with three repeats leads to a decrease in MAOA activity resulting in an increased serotonin level in the synapses, which was reported to increase the risk of aggression and antisocial behavior [34].

The results of *MAOA* genotyping presented in Table 3 show that only variants with 3 or 4 repeats (3R or 4R) could be detected in the investigated groups of patients. In patients with an unfavorable PC prognosis, the 3R variant tended to be slightly more common (OR = 1.582), however the difference between the groups failed to reach statistical significance.

**Table 3:** Genotype frequencies for the MAOA-μVNTR 30 bp polymorphism of the MAOA in groups of PC patients defined as ‘polar opposites’ prognostically

Polymorphism *MAOA-μVNTR* 30 bp, number of repeats	Disease prognosis (according to PSADT)				OR (95% CI^*^)	*P*-value
	Unfavorable (low PSADT)		Favorable (high PSADT)			
	*n*	%	*n*	%		
4R	16	55.17	37	66.07	0.632 (0.252–1.582)	0.327
3R	13	44.83	19	33.93	1.582 (0.632–3.960)	0.327
Total	29^**^	100.0	56^**^	100.0	-	-

^*^CI – confidence interval. ^**^Analyzed patient numbers are lower than group totals as genotyping failed in some cases.

#### *COMT* gene

The *COMT* gene encodes catechol-O-methyltransferase (COMT), an enzyme that breaks down dopamine in the prefrontal cortex of the human brain. The Val158Met polymorphism of the gene that leads to a decreased COMT activity in Met^158^ allele bearers (especially A/A homozygotes) was reported to be linked with neuropsychiatric conditions, such as alcohol dependence, bipolar disorder, schizophrenia [35–37]. The results presented in Table 4 show that the heterozygosity (G/A genotype) for this locus is slightly overrepresented in patients with unfavorable PC prognosis. In contrast, the homozygosity at the locus and, especially, the presence of the G/G genotype seemed to be weakly associated with a better prognosis of the disease. At the same time, PC prognosis did not depend on the presence of the genotypes defining either normal or reduced COMT activity. None of the observed minor differences in genotype frequencies reached statistical significance.

**Table 4:** Genotype frequencies for the Val158Met polymorphism of the COMT gene in groups of PC patients defined as ‘polar opposites’ prognostically

Polymorphism *COMT* Val158Met	Disease prognosis (according to PSADT)				OR (95% CI^*^)	*P*-value
	Unfavorable (low PSADT)		Favorable (high PSADT)			
	*n*	%	*n*	%		
G/G	3	12.5	11	21.57	0.519 (0.130–2.068)	0.353
G/A	15	62.5	27	52.94	1.481 (0.549–3.997)	0.438
A/A (reduced COMT activity)	6	25.0	13	25.49	0.974 (0.318–2.981)	0.964
*G/G or G/A (normal COMT activity)*	*18*	*75.0*	*38*	*74.5*	*1.026 (0.335–3.140)*	*0.964*
*Homozygosity*	*9*	*37.5*	*24*	*47.06*	*0.675 (0.250–1.821)*	*0.438*
Total	24^**^	100.0	51^**^	100.0	-	-

^*^CI – confidence interval. ^**^Analyzed patient numbers are lower than group totals as genotyping failed in some cases. *Additional genotype groupings are italicized.*

#### *DRD4* gene

The dopamine receptor gene *DRD4* is being intensively studied with regard to its possible involvement in several mental disorders, such as attention deficit disorder (ADHD), gambling addiction, alcoholism, drug addiction, and schizophrenia [30, 38]. The sequence of this gene contains a variable number of 48 bp tandem repeats (VNTR) in the third exon, ranging from 2 to 11 repeats (2R to 11R). The 4R allele is the most common in many populations, while the frequencies of the 2R and 7R alleles vary widely [39]. Test results for this polymorphism are shown in Table 5, which demonstrates that the presence of homozygosity at this VNTR locus, especially the 2R/2R genotype (OR = 0.218) appeared to be associated with a better prognosis, whereas the heterozygosity tends to be overrepresented in PC patients with a poorer prognosis (OR = 2.333). Unfortunately, the number of analyzed cases was very low due to technical problems, and the differences between the groups were not statistically significant.

**Table 5:** Genotype frequencies for the DRD4 gene VNTR 48 bp polymorphism in groups of PC patients defined as ‘polar opposites’ prognostically

Polymorphism *DRD 4* VNTR 48 bp, number of repeats	Disease prognosis (according to PSADT)				OR (95% CI^*^)	*P*-value
	Unfavorable (low PSADT)		Favorable (high PSADT)			
	*n*	%	*n*	%		
2R/2R	1	5.0	7	19.44	0.218 (0.025–1.917)	0.170
2R/4R	4	20.0	2	5.56	4.250 (0.704–25.670)	0.115
3R/3R	2	10.0	2	5.56	1.889 (0.245–14.550)	0.541
3R/4R	0	0	1	2.78	0.577 (0.022–14.835)	0.740
4R/4R	8	40.0	17	47.22	0.745 (0.246–2.257)	0.603
4R/5R	2	10.0	2	5.56	1.889 (0.245–14.550)	0.541
4R/6R	2	10.0	2	5.56	1.889 (0.245–14.550)	0.541
4R/7R	0	0	1	2.78	0.577 (0.022–14.893)	0.740
6R/6R	1	5.0	2	5.56	0.895 (0.076–10.528)	0.929
*Homozygosity*	*12*	*60.0*	*28*	*77.8*	*0.429 (0.130–1.410)*	*0.163*
*Heterozygosity*	*8*	*40.0*	*8*	*22.2*	*2.333 (0.709–7.675)*	*0.163*
Total	20^**^	100.0	36^**^	100.0	-	-

^*^CI – confidence interval. ^**^Indicated numbers of analyzed patients differ from group totals as genotyping failed in some cases. *Additional genotype groupings are italicized.*

The –521 C/T polymorphism in the promoter region of the *DRD4* gene influences its transcription level (decreased by 40% in T/T homozygotes) [40], and the presence of the C allele was previously shown to be associated with schizophrenia development risk [38, 40], whereas the T allele was found to predispose to heroin addiction [41]. Table 6 presents the results that show a non-significant trend for the presence of the C allele at the locus to be associated with a more favorable PC prognosis.

**Table 6:** Genotype frequencies for the −521 C/T DRD4 gene polymorphism in groups of PC patients defined as ‘polar opposites’ prognostically

Polymorphism *DRD 4* −521 C/T	Disease prognosis (according to PSADT)				OR (95% CI^*^)	*P*-value
	Unfavorable (low PSADT)		Favorable (high PSADT)			
	*n*	%	*n*	%		
C/C	4	12.9	10	16.13	0.770 (0.221–2.687)	0.682
C/T	11	35.48	27	43.55	0.713 (0.293–1.737)	0.457
T/T (reduced gene transcription)	16	51.61	25	40.32	1.579 (0.663–3.761)	0.303
*C/C or C/T (normal gene transcription)*	*15*	*48.39*	*37*	*59.68*	*0.633 (0.266–1.509)*	*0.303*
*Homozygosity*	*20*	*67.74*	*35*	*56.45*	*1.403 (0.576–3.418)*	*0.457*
Total	31^**^	100.0	62^**^	100.0	-	-

^*^CI – confidence interval. ^**^Indicated numbers of analyzed patients differ from group totals as genotyping failed in some cases. *Additional genotype groupings are italicized.*

## DISCUSSION

The small pilot study described in this paper tentatively suggests that there may be an association between a few polymorphic variants of genes controlling neurotransmitter metabolism and the prognosis of PC. This previously unexplored association is especially intriguing as all the four polymorphic genes included in the analysis are known to modulate the predisposition to a range of neuropsychiatric disorders [28–35, 37, 38].

We believe that the most interesting results within our study were obtained for the STin2 polymorphism of the serotonin transporter gene *SLC6A4*. It is well documented that disturbances in the serotonin-controlled neuromodulation system involving the serotonin transporter are implicated in the pathogenesis of many behavioral disorders (anxiety, depression, obsessive compulsive disorder, aggression, addiction, suicide) as well as in major psychiatric conditions, such as bipolar disorder and schizophrenia [42, 43]. Regarding the *SLC6A4* gene, the homozygous genotype 12R/12R at the STin2 polymorphic locus was repeatedly reported to be linked with a predisposition to schizophrenia development [42, 44], whereas the 12R/10R heterozygosity appeared to be protective [44]. Interestingly, our results show that PC patients with an unfavorable disease prognosis tend to have the 12R/10R genotype more often (OR = 2.278; 95% CI = 0.953–5.444). Conversely, the 12R/12R genotype and the homozygosity for the locus (comprising the 12R/12R and 10R/10R genotypes) appeared to be associated with a better PC prognosis (OR = 0.431; 95% CI = 0.176–1.057 for all homozygotes). Although our results were marginally non-significant statistically due to limited numbers of analyzed cases, these observations are perfectly in line with earlier communications reporting a decreased risk of developing PC amongst schizophrenia patients [16–18]. Indeed, the reported overrepresentation of the 12R/12R genotype in schizophrenia patients [44] may possibly create a ‘PC-protective genetic background’ explaining a reduced PC incidence in men suffering from this psychiatric condition.

The results obtained for the other polymorphisms assessed in this study were in general inconclusive, but a few observations deserve to be noted. Monoamine oxidase A (MAOA) encoded by the *MAOA* gene is another enzyme involved in serotonin catabolism, and the 30 bp VNTR in the promoter region of this gene is functionally important as the presence of its allele with four repeats elevates *MAOA* gene transcription level resulting in an increased enzyme activity [33, 45]. Studies by other authors indicated that the lack of MAOA activity caused by the presence of the VNTR allele with three repeats is often associated with an increased risk of aggressivity and antisocial behavior [34]. Our results show that the same allele tends to be slightly overrepresented in PC patients with unfavorable disease prognosis, however the association failed to reach statistical significance. This result is not surprising as no influence of the major *MAOA-μVNTR* variants on PC risk or prognosis could be found by a properly powered study of White et al. [46].

The COMT is one of the enzymes regulating the central dopamine function. The presence of the MET^158^ allele of the *COMT* gene reduces COMT enzyme activity and was previously reported both to predispose to psychiatric conditions [33–35] and to be possibly implicated in cancer risk modulation [47]. However, the outcome of our study has not revealed any significant effect of this *COMT* polymorphism on PC prognosis. This negative finding corroborates results previously published by other authors [48].

The D_4_ dopamine receptor encoded by the *DRD4* gene, which is highly polymorphic, is one of D_2_-like transmembrane dopamine receptors controlling the G-protein-mediated signaling pathway through adenylyl-cyclase inhibition [49]. The two *DRD4* polymorphisms analyzed in our study were repeatedly shown to be associated with a range of psychiatric disorders [30, 38], but there was no pre-existing information on possible links between these gene variants and cancer. According to previously published reports, the *DRD4* VNTR variants with a higher number of repeats tended to predispose to depression and other mental disorders [30]. It is remarkable that among our patients, the genotype 2R/2R with the lowest number of repeats appeared to be linked with a better PC prognosis (OR = 0.218; 95% CI = 0.025–1.917), but the difference failed to reach statistical significance. Likewise, the presence of the homozygosity at this VNTR locus tended to be protective, whereas the heterozygosity was slightly overrepresented among PC patients with unfavorable prognosis (see Table 5). Another *DRD4* gene variant demonstrating a weak association with a better PC prognosis was the presence of the C allele at position -521 of *DRD4* gene promoter. Although this association was not statistically significant, it is noteworthy that the same allele is associated with an increased risk of developing schizophrenia [38, 40]. Therefore, its presence may potentially contribute to the ‘PC-protective genetic background’ probably existing in patients with schizophrenia and discussed above in relation to the *SLC6A4* gene STin2 polymorphism.

It should be accepted that this small pilot study had serious limitations. Small study size restricted its statistical power, and technical problems that in most cases could be attributed to DNA degradation during long-term sample storage, further limited the number of successfully performed tests. For this reason, it was difficult to expect obtaining statistically significant differences between genotype-defined subgroups and results often looked inconclusive. Nevertheless, some interesting trends could be revealed, especially those probably related to the inverse relationship between risks of developing schizophrenia and PC.

Oncological conditions are known to occur less frequently in schizophrenia patients [11, 13–16], and this phenomenon primarily affects men [50], being especially pronounced for PC [13, 16–18]. Interestingly, PC has recently emerged as a cancer, development of which strongly depends on neurogenic regulatory pathways provided by nerves growing as an important TME component [51]. It is also notable that schizophrenia-related alterations in neurotransmitter-modulated tumor angiogenesis were recently hypothesized to be a factor reducing neoplasia development risk in this group of patients [52]. Furthermore, neurotransmitters, such as serotonin and dopamine, are now regarded as major factors modulating neoplastic growth through influences on angiogenesis and neoplastic cell proliferation [53]. The results presented in this paper indicate that the presence of certain polymorphic variants of the *SLC6A4* and *DRD4* genes related to serotonin and dopamine signaling pathways respectively appears to correlate with PC prognosis. Although our findings remain inconclusive, these preliminary observations deserve to be explored in depth. Further larger studies are needed for clarifying the role of neurotransmitter metabolism gene polymorphisms in PC pathogenesis.

## MATERIALS AND METHODS

### Selection of PC patients for genotyping

This study was designed with the aim of comparing selected polymorphic gene variant presence in PC patients with either very favorable or utterly unfavorable variants (i. e. ‘polar opposites’) of disease prognosis defined by PSADT values measured before treatment initiation. The PSADT was calculated using an online calculator, in accordance with the accepted recommendations of the Memorial Sloan Kettering Cancer Center [54]. Prognostically favorable cases (*N* = 67) were defined by the PSADT values exceeding 30 months, while a prerequisite for inclusion was a PSA history of at least one year. In some of these patients, the PSADT values could not be determined as there was no PSA concentration increase throughout the observation period. In contrast, cases where the PSADT values did not exceed two months (*N* = 40) were regarded as prognostically unfavorable.

### Genotyping

The most significant polymorphisms in four genes encoding regulatory proteins controlling neurotransmitter metabolism were selected for the study: the serotonin transporter gene (*SLC6A4)*, the catechol-ortho-methyltransferase gene (*COMT*), the monoamine oxidase A gene (*MAOA*), the dopamine receptor gene (*DRD4*). All these genes are known to be associated with mental disorders: schizophrenia, depression, deviant behavior. The distribution of gene polymorphisms was assessed and compared in two groups of patients with PC characterized as ‘polar opposites’ in terms of disease prognosis.

Genomic DNA was extracted from peripheral blood cells using the reagent kit “DNA-Extran-1”, (Syntol, Russia) in accordance with the manufacturer’s instructions. The isolated DNA was used for the detection of the selected gene polymorphisms by PCR.

Determination of polymorphism in STin2 VNTR of the *SLC6A4* gene was carried out using the method of polymerase chain reaction (PCR). The reagent kit “Encyclo Plus PCR kit” and a pair of sequence-specific primers were employed. The primers synthesized by Eurogen (Russia) had the following sequences:

F 5′- CAATGTCTGGCGCTTCCCCTACATAT -3′

R 5′- GACATAATCTGTCTTCTGGCCTCTCAAG -3′

The following PCR conditions were applied for the detection of the STin2 VNTR: Initial denaturation at 95°C for 4 min was followed by 31 three-step cycles that comprised denaturation for 30 seconds at 95°C, annealing for 30 seconds at 67°C, and extension for 40 seconds at 72°C. At the last stage, the final extension step was performed at 72°C for 5 minutes.

PCR results were assessed using DNA gel electrophoresis in 12% polyacrylamide gel, followed by staining with SYBR Green I (Lumiprobe, Russia) and visualization of the fragments in transmitted with 100 bp DNA ladder (SibEnzyme, Russia) used for fragment length determination.

The amplified fragments were distributed as follows: 222 bp - 9 repeats, 261 bp - 10 repeats, 300 bp - 12 repeats.

The PCR used for the determination of *MAOA-μVNTR* polymorphism was carried out using the reagent kit “Encyclo Plus PCR kit” and a pair of primers synthesized by Eurogen (Russia).

F 5′-ACAGCCTGACCGTGGAGAAG-3′

R 5′-GAACGGACGCTCCATTCGGA-3′

The following PCR conditions were applied for the detection of the *MAOA-μVNTR*:

Initial denaturation at 95°C for 3 min was followed by 30 three-step cycles that comprised denaturation for 30 seconds at 95°C, annealing for 30 seconds at 60°C, and extension for 40 seconds at 72°C. At the last stage, the final extension step was performed at 72°C for 5 minutes.

The method used for PCR result visualization did not differ from that used for STin2 VNTR and described above. The amplified fragments were distributed as follows:

291 bp - 2 repeats, 321 bp - 3 repeats, 336 bp - 3.5 repeats, 351 bp - 4 repeats, 381 bp - 5 repeats.

To analyze the polymorphic Val158Met locus (472A>G, rs4680) of the *COMT* gene and the *DRD4* locus rs1800955 (-521 C/T promoter polymorphism), we used the SNP-express-RV reagent kits (Litekh, Russia) and SNP-Screen (Syntol, Russia), respectively, by real-time PCR using CFX96 Touch™ Real-Time PCR Detection System (Bio-Rad, USA) according to the manufacturer’s instructions.

Determination of *DRD4* polymorphism (VNTR 48bp) was carried out using the “TAPOTILI - locus DRD4-VNTR” reagent kit (TAPOTILI company, Russia) by real -time PCR using CFX96 Touch™ Real-Time PCR Detection System (Bio-Rad, USA) according to the manufacturer’s instructions.
